# Population Pharmacokinetics and Dose Optimization of Ganciclovir in Critically Ill Children

**DOI:** 10.3389/fphar.2020.614164

**Published:** 2021-01-18

**Authors:** SiChan Li, Chang Shu, SanLan Wu, Hua Xu, Yang Wang

**Affiliations:** ^1^ Department of Clinical Pharmacy, Wuhan Children's Hospital, Tongji Medical College, Huazhong University of Science and Technology, Wuhan, China; ^2^ Department of Pharmacy, Union Hospital, Tongji Medical College, Huazhong University of Science and Technology, Wuhan, China

**Keywords:** ganciclovir, population pharmacokinetics, children, dosing, critically ill

## Abstract

**Objective:** The present study aims to establish a population pharmacokinetic model of ganciclovir and optimize the dosing regimen in critically ill children suffering from cytomegalovirus related disease.

**Methods:** A total of 104 children were included in the study. The population pharmacokinetic model was developed using the Phoenix NLME program. The final model was validated by diagnostic plots, nonparametric bootstrap, visual predictive check, and normalized prediction distribution errors. To further evaluate and optimize the dosing regimens, Monte Carlo simulations were performed. Moreover, the possible association between systemic exposure and hematological toxicity were also monitored in the assessment of adverse events.

**Results:** The ganciclovir pharmacokinetics could be adequately described by a one-compartment model with first-order elimination along with body weight and estimated glomerular filtration rate as significant covariates. As showed in this study, the typical population parameter estimates of apparent volume of distribution and apparent clearance were 11.35 L and 5.23 L/h, respectively. Simulations indicated that the current regimen at a dosage of 10 mg/kg/d would result in subtherapeutic exposure, and elevated doses might be required to reach the target ganciclovir level. No significant association between neutropenia, the most frequent toxicity reported in our study (19.23%), and ganciclovir exposure was observed.

**Conclusion:** A population pharmacokinetic model of intravenous ganciclovir for critically ill children with cytomegalovirus infection was successfully developed. Results showed that underdosing of ganciclovir was relatively common in critically ill pediatric patients, and model-based approaches should be applied in the optimizing of empiric dosing regimens.

## Introduction

Ganciclovir (GCV) is a pro-drug nucleoside guanosine analogue that exhibits potent activity against herpesviruses, including cytomegalovirus (CMV) (Villarreal, 2001). After phosphorylation in CMV infected cells, GCV is transformed into its triphosphate derivative, which is the active product that inhibits viral replication. Currently, GCV is not only approved for the treatment and prevention of CMV infections in immunocompromized patients (Sia and Patel, 2000), but also the treatment of congenital CMV infection and other CMV related diseases as an off-label drug.

As previous study showed that the oral bioavailability of GCV was less than 10% (Boeckh et al., 1998), despite the fact that the co-administration of food would increase its absorption. Hence, intravenous infusion was the main method to deliver GCV. However, following intravenous infusion, GCV was weakly bounded to plasma proteins (1–2%) over a concentration of 0.5–51 mg/L (McGavin and Goa, 2001), and it could easily penetrate the cerebrospinal fluid. Several studies showed that a large portion of the administered dose was eliminated from the body by glomerular filtration and renal tubular secretion as unchanged drug, which exhibited a good correlation between the clearance of GCV and creatinine clearance in adult patients (Roberts et al., 2014b; Al-Badr and Ajarim, 2018). As previous pharmacokinetic/pharmacodynamic studies confirmed, the desirable antiviral outcomes would require an area under drug plasma concentration-time curve over 24 h (AUC_0-24_) of 40–50 μg h/ml in both pediatric and adult patients following solid organ transplant (Wiltshire et al., 2005; Dong et al., 2018). However, it was estimated that nearly 80% patients may fail to achieve the target AUC level using the current pediatric GCV dosing regimen, thus increasing the risk of therapeutic failure in pediatric patients (Stockmann et al., 2015).

In addition, the pharmacokinetic profiles of GCV were highly variable among pediatric patients, especially among hospitalized children with critical illness. A growing evidence showed that altered pharmacokinetic characteristics in critically ill children caused by pathophysiological changes might reduce the likelihood of attaining pharmacodynamic target in this population (Roberts and Lipman, 2009; Roberts et al., 2014a). Furthermore, more studies found that CMV reactivation was prevalent in immunocompetent critically ill patients with a high incidence of 15–20%. Worse still, CMV reactivation was also considered to be correlated with clinical adverse outcomes and the increase of inpatient mortality (Limaye et al., 2008; Papazian et al., 2016; Alyazidi et al., 2018).

Therefore, the aims of the present study were to develop a population pharmacokinetic (PopPK) model for critically ill children receiving intravenous GCV and to further evaluate and optimize the current dosing regimen in this vulnerable population based on modeling and simulating approaches.

## Methods

### Study Design and Patient Population

This trial was an open-labeled, retrospective pharmacokinetic study of GCV, conducted in Wuhan Children's hospital from Dec 2017 to Jan 2020. Critically ill patients aged one month to 18 years with confirmed CMV infection who had received intravenous GCV were included in our study. While for patients who are allergic to GCV, lessing than 24 h of GCV therapy, missing data for key variables, or patients simultaneously enrolled in another clinical trial were excluded.

This study was designed in accordance with legal requirements and the Declaration of Helsinki and was approved by the Ethics Committee of Wuhan Children's hospital with waiving of the need for informed consent (approval number: 2020R075-E01).

### Dosing Regimen, Pharmacokinetic Sampling and Data Collection

GCV was administered by intravenous infusion over 1 h at a dose of 5 mg/kg twice a day. An opportunistic sampling strategy was adopted (Leroux et al., 2015). The residual serum samples were drawn from routine biochemical specimens and stored at −20°C until assay. Serum concentrations were tested within 48 h after sampling. The actual administration time and sampling time of each sample were precisely recorded and used in PopPK analysis.

Demographic and physiological characteristics of all patients were obtained from the electronic medical records system, including gender, age, body weight (WT), height, blood urea nitrogen (BUN), serum creatinine concentration (SCR), uric acid (UA), total bilirubin concentration (TBIL), alanine aminotransferase concentration (ALT), and aspartate aminotransferase concentration (AST). The estimated glomerular filtration rate (eGFR) and body surface area (BSA) were calculated based on the Gao formula (Gao et al., 2013) and the Mosteller formula (Mosteller, 1987), respectively. And based on the calculated data, the renal function status were classified into (1) elevated renal function (eGFR ≥120 mL/min/1.73m^2^), (2) normal renal function (90 mL/min/1.73m^2^ ≤ eGFR <120 mL/min/1.73m^2^), (3) mild renal insufficiency (60 mL/min/1.73m^2^ ≤ eGFR <90mL/min/1.73m^2^), (4) moderate renal insufficiency (30 mL/min/1.73m^2^ ≤ eGFR <60mL/min/1.73m^2^), (5) severe renal insufficiency (eGFR <30 mL/min/1.73 m^2^).

### Analytical Method of Ganciclovir

GCV concentrations were quantified using a validated high-performance liquid chromatography (HPLC Agilent Technologies Inc., 1260 infinity Ⅱ) with ultraviolet (UV) detection. Sample preparation was carried out using C18 solid-phase extraction columns (Agela Technologies, Cleanert ODS C18, 500 mg/3 mL). A 0.5-ml volume of serum sample was pipetted into a column preconditioned with methanol and water, then the analytes were eluted with 1 mL of 20% methanol. The chromatographic separation was performed using methanol (4%) and water (96%) as the mobile phase in a DIKMA Luster C18 column (5 μm, 4.6 × 250 mm) at 30°C. The flow rate was 0.8 ml/min. Samples were then detected at 254 nm. The calibration curve was linear over a concentration range of 0.1–20.0 μg/mL, and the lower limit of quantification (LLOQ) was 0.1 μg/mL. The intra- and inter-day coefficients of variation were less than 8%.

### PopPK Modeling

Pharmacokinetic data of GCV was analyzed using the Phoenix® NLME software (Version 8.1, Pharsight Corporation, USA). For statistical analysis and output visualization, RStudio (version 1.3, http://www.rstudio.com/) was employed. Lindstrom-Bates First-Order Conditional Estimation (FOCE-LB) algorithm was applied in all model runs.

### Base Model

Both one- and two-compartment models with first-order elimination were tested to fit the GCV concentration data. The initial structural model was selected on the basis of visual inspection of the data and the values of Akaike information criterion (AIC) and Bayesian information criterion (BIC).

The interindividual variability was modeled for each pharmacokinetic parameter using an exponential model (Eq. 1).

$$P_{i}=\theta \times \exp\left(\eta_{i}\right)$$ (1)

where *P*_*i*_ denotes the estimated pharmacokinetic parameter for individual *i*, θ is the population typical value of the parameter, and η_*i*_ denotes the random variable for individual *i*, which is defined as normally distributed with a mean of 0 and a variance of *ω* ^2^.

Additionally, proportional and combined-error models were explored to estimate the residual error variability. The equations were as follows (Eqs 2–4).

Y=IPRED+ε (2)

Y=IPRED×(1+ε) (3)

$$Y=\mathrm{IPRED}\times \left(1+\varepsilon_{1}\right)+\varepsilon_{2}$$ (4)

where *Y* and IPRED denote the measured concentration and individual prediction, respectively. And *ε* devotes the residual random error, which is assumed to be Gaussian distributed with a mean of 0 and a variance of *σ* ^2^.

### Covariate Analysis

Demographic data (gender, age, WT, height, BSA), renal function (BUN, SCR, UA), and hepatic function (TBIL, ALT, AST) were investigated as potential covariates for their influences on the pharmacokinetics of GCV. Besides, kidney function (KF) was also taken into account as a dimensionless parameter, and the value of which was calculated as dividing individual eGFR by the normal renal function (120 mL/min/1.73 m^2^). Prior to performing covariate screening, correlation coefficients were calculated for all pair-wize variables, meanwhile highly intercorrelated covariates (correlation coefficient >0.5) were not simultaneously introduced into the model.

Covariates analysis was carried out by means of a stepwise forward inclusion and backward elimination method. And covariates were screened by implementing a likelihood ratio test on the changes in the objective function value (OFV). During the forward selection, a significant reduction in OFV of 3.84 or more (*p* < 0.05) was considered sufficient for inclusion in the base model. This process was repeated until the full model has been constructed. Then, backward elimination was performed to remove covariates from the full model. And an increase in OFV of at least 6.63 (*p* < 0.01) was required to retain the covariate in the final model. Meanwhile, the biological plausibility and clinical significance of the potential covariates were also considered.

Relationships between potential variables and pharmacokinetic parameters were assessed. Power model and exponential model were used to evaluate the continuous covariates and categorical covariates, respectively. Furthermore, to describe differences in body size and the processes of clearance maturation, both WT and BSA were tested using theory-based allometric models. The description of seven candidate models (Model Ⅰ-Ⅶ) were summarized in Table 1.

**TABLE 1:** Model description of the seven candidate models for clearance.

**Candidate models**	**Model description**		
		***k*_1_**	**MF**
Model Ⅰ: the 3/4 allometric model		0.75	1
Model Ⅱ: the simplest WT-based exponent model		Estimated	1
Model Ⅲ: the simplest BSA-based exponent model		Estimated	1
Model Ⅳ: the maturation model	$\text{CL}/\text{F}=\theta_{\text{CL}}\times {\left(\frac{\text{WT}}{{\text{WT}}_{\text{median}}}\right)}^{k1}\times \text{MF}$	0.75	$M\text{F}=\frac{1}{1+{\left(\frac{\text{Age}}{{\text{TM}}_{50}}\right)}^{-\gamma }}$
Model Ⅴ: the WT-dependent exponent model		$k_{1}=k_{0}-\frac{k_{\max}\times {\text{WT}}^{\gamma }}{k_{50}^{\gamma }+{\text{WT}}^{\gamma }}$	1
Model Ⅵ: the age-dependent exponent model		$k_{1}=k_{0}-\frac{k_{\max}\times {\text{Age}}^{\gamma }}{k_{50}^{\gamma }+{\text{Age}}^{\gamma }}$	1
Model Ⅶ: the BSA-dependent exponent model	$\text{CL}/\text{F}=\theta_{\text{CL}}\times {\left(\frac{\text{WT}}{{\text{WT}}_{\text{median}}}\right)}^{k1}\times \text{MF}$	$k_{1}=k_{0}-\frac{k_{\max}\times {\text{BSA}}^{\gamma }}{k_{50}^{\gamma }+{\text{BSA}}^{\gamma }}$	1

θ_CL_, typical value of clearance; θ_Vd_, typical value of volume of distribution; MF, factor for maturation; TM_50_, maturation half-time; γ, Hill coefficient defining the steepness of the sigmoidal curve; k^1^, allometric exponent; k_0_, the exponent at a theoretical weight of 0 kg , BSA of 0 m^2^, or age at 0 years; k_max_, a maximum decrease of the exponent; k_50_, the weight, BSA or age when a 50% drop in the maximum decrease of the exponent is achieved.

### Model Validation

The final model was validated both graphically and statistically by goodness-of-fit plots, nonparametric bootstrap analysis, visual predictive check (VPC), and normalized prediction distribution errors (NPDEs). The goodness-of-fit was evaluated using diagnostic plots, including observed concentrations (DV) vs. population predictions (PRED), DV vs. individual predictions (IPRED), conditional weighted residuals (CWRES) vs.. PRED, and CWRES vs. time. The nonparametric bootstrap approach was utilized to generate 1,000 re-sampled datasets, among which the median estimates with 95% confidence intervals (CIs) were further calculated and compared with the final parameter estimates, to assess the precision of the final model. The VPC was performed using 1,000 simulations to assess the predictive performance of the final model. The observations and simulations were then compared by computing the 25th, 50th, and 97.5th percentiles for each. The model was further evaluated using statistical tests and visual inspection of NPDE plots, including quantile-quantile plot, histogram of the NPDE distribution, scatterplots of NPDE vs. PRED and NPDE vs. time after the last dose.

### Dosing Simulations

A Monte Carlo simulation with 10,000 iterations was performed to evaluate and optimize the dosing regimens using the pharmacokinetic parameter estimates obtained from the final model. Concentration vs. time profiles of various dosage regimens in patients with different levels of renal function and WT were simulated. Meanwhile, the AUC_0-24_ values of each simulated patient were also computed. The probability of target attainment (PTA) of an AUC_0-24_ of ≥40 μg h/ml was subsequently determined. As to the dosage regimen, it was considered acceptable if the PTA is higher than 80%.

### Assessment of Adverse Events

Potential adverse effects were closely monitored in the study. Hematological toxicities were evaluated by a comparison of hematological parameters, including neutrophil count, platelet count, lymphocyte count, and hemoglobin concentration, obtained before and after the administration of GCV. Neutropenia, thrombocytopenia, lymphopenia, and anemia were defined and graded according to the Common Terminology Criteria for Adverse Events (CTCAE) v5.0 (National Cancer Institute, 2017). Statistical analyses were carried out using SPSS software Version 19.0 (SPSS Inc.,Chicago, IL, USA).

## Result

### Patient Characteristics

**FIGURE 1:**
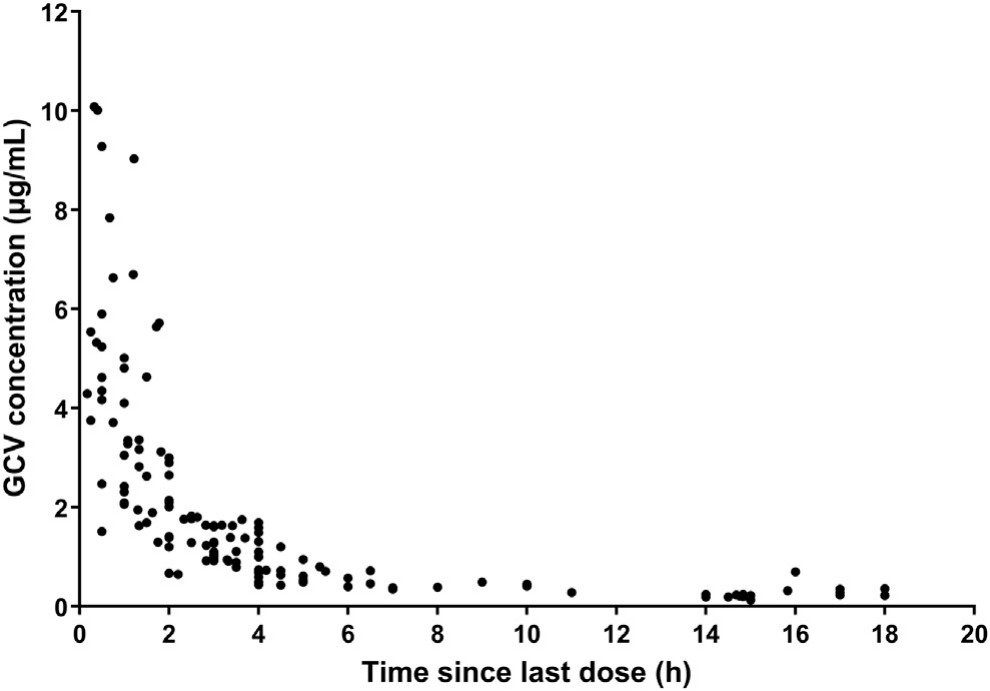
GCV concentrations vs. time.

A total of 104 patients were enrolled in the present study, and 138 measured GCV concentrations (range 0.13–10.08 μg/mL) were obtained from 1–3 samples per patient. No patient was excluded according to the exclusion criteria. The GCV concentration-versus-time profile was showed in Figure 1. The study population consisted of 54 male and 50 female patients between 0.10 and 12.83 years old. And the body weights were recorded from 2.5 to 55.0 kg. Among all subjects, 64 (61.5%) of these patients were infants aged 0–3 years, 28 (26.9%) were young children aged 3–6 years, and twelve (11.5%) were old children aged >6 years. According to the criteria described previously, 12 patients were classified as the elevated renal function group, 80 patients as the normal renal function group, and 11 patients as the mild renal insufficiency group, while one patient with severe renal insufficiency was excluded from grouping and subgroup comparison. The demographic and physiological characteristics of patients were summarized in Table 2.

**TABLE 2:** Demographic and physiological characteristics of patients in this study (*n* = 104).

	Number	Mean ± SD	Median (range)
Patients	104		
Gender (M**:**F)	54:50		
Age (years)		3.06 ± 2.99	2.46 (0.10–12.83)
WT (kg)		13.7 ± 8.3	12.0 (2.5–55.0)
Height (cm)		90.8 ± 25.3	90.0 (44.0–161.0)
BSA (m^2^)		0.58 ± 0.25	0.55 (0.17–1.57)
GCV dose (mg·kg^−1^·d^−1^)		9.7 ± 0.7	10.0 (5.6–12.2)
GCV concentration (μg•mL^−1^)		2.11 ± 2.16	1.39 (0.13–10.08)
Laboratory parameter			
BUN (mmol/L)		3.11 ± 1.18	2.93 (0.77–6.60)
UA (μmol/L)		252.4 ± 89.7	244.3 (86.0–522.0)
SCR (μmol L^−1^)		26.9 ± 8.3	26.0 (12.7–68.4)
eGFR (mL/min/1.73 m^2^)		106.96 ± 15.39	110.85 (14.61–129.13)
TBIL (μmol L^−1^)		13.9 ± 21.5	7.5 (2.2–169.7)
ALT (U L^−1^)		62.5 ± 81.1	25.0 (6.0–440.0)
AST (U L^−1^)		68.9 ± 60.6	50.5 (9.0–442.0)

WT, body weight; BSA, body surface area; GCV, ganciclovir; BUN, blood urea nitrogen; UA, uric acid; SCR, serum creatinine concentration; eGFR, estimated glomerular filtration rate; TBIL, total bilirubin concentration; ALT, alanine aminotransferase concentration; AST, aspartate aminotransferase concentration.

### PopPK Model Development

In the present study, the two-compartment model yielded a similar OFV as compared to the one-compartment model. In consideration of the results gathered from published papers and the clinical practicality of the model, a one-compartment model with first-order elimination was deemed as an appropriate structural model. And the apparent clearance (CL) and apparent volume of distribution (*V*_d_) was then derived from the PopPK model. The inter-individual variability was optimally described by an exponential model, while the residual variability could be best expressed using a proportional model.

**FIGURE 2:**
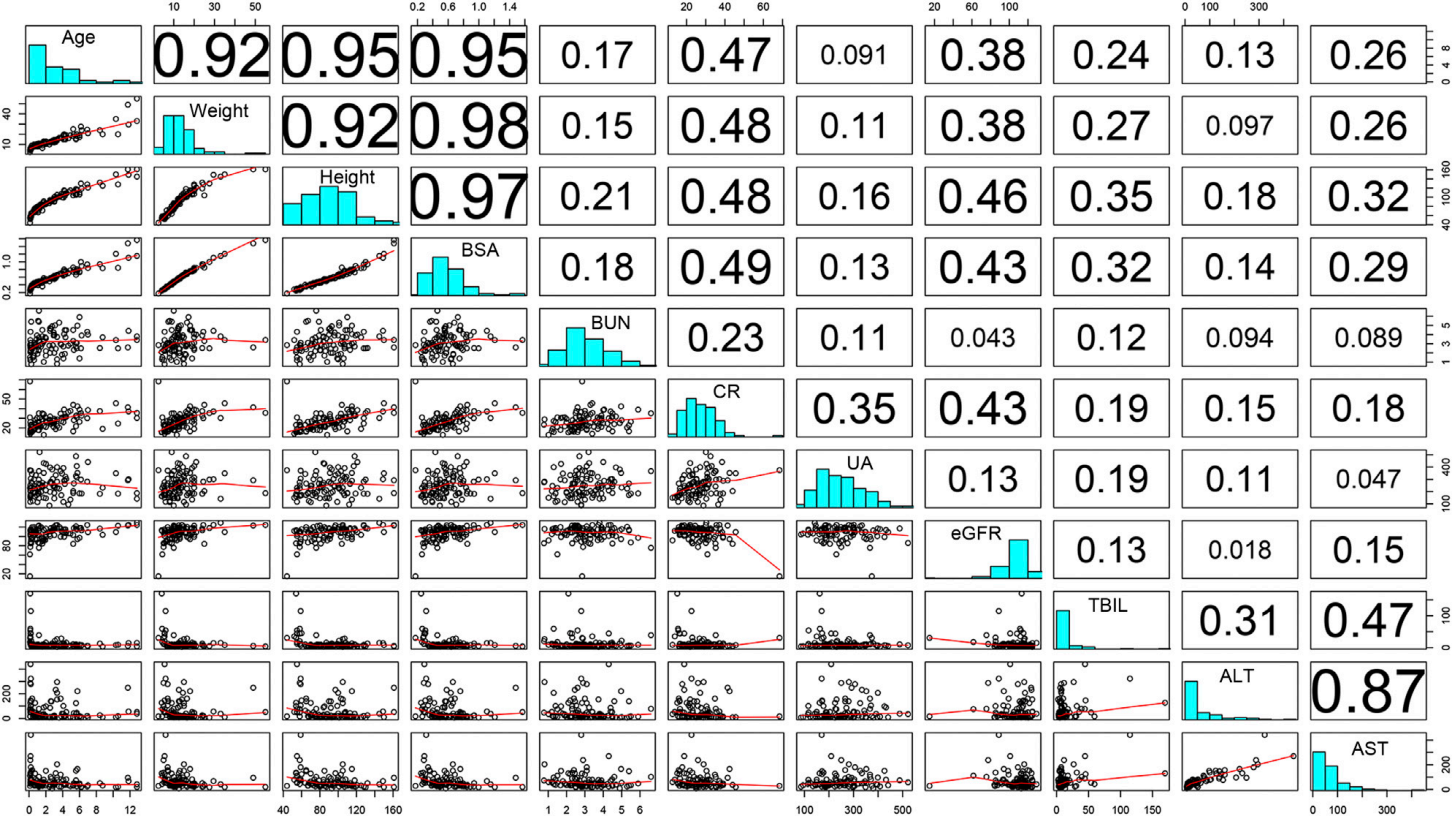
Scatterplot matrix of covariate analysis.

In Figure 2, the resulting matrix of correlation coefficients among the potential covariates is visualized. Under the premise that simultaneous introduction of collinear variables was avoided, the covariates were added to the base model to construct a full model. To account for the influence of developmental changes, WT- or BSA-based allometric models (Model Ⅰ-Ⅶ) for CL were tested, as presented in Table 3. Among the candidate models, the simplest WT-based exponent model (Model Ⅱ) corresponding to the lowest OFV, AIC, and BIC, respectively, was chosen for further analysis. After the covariate screening procedure, WT and KF were identified as determinant variables for CL, and were also related to significant drops in the OFV of 58.22 points and 7.92 points, respectively. Besides, WT had a notable effect on *V*_d_, which significantly reduced the OFV by 8.05 units. The covariate screening procedure according to the descending order of OFV was detailed in Table 4.

**TABLE 3:** Parameter estimates of the seven candidate models for clearance.

Parameters	Model I: the 3/4 allometric model	Model II: the simplest WT-based exponent model	Model III: the simplest BSA-based exponent model	Model IV: the maturation model	Model V: the WT-dependent exponent model	Model VI: the age-dependent exponent model	Model VII: the BSA-dependent exponent model
OFV	311.74	298.25	312.02	306.98	332.86	298.85	309.25
AIC	323.74	308.25	324.02	320.98	350.86	316.85	327.25
BIC	341.31	322.89	341.58	341.47	377.2	343.19	353.59
θ_CL_ (SE%)	4.68 (5.77)	4.57 (6.21)	4.68 (6.21)	10.05 (7.73)	4.35 (8.62)	5.62 (7.38)	6.53 (10.29)
θ_Vd_ (SE%)	11.18 (8.90)	10.97 (9.11)	10.99 (9.14)	14.05 (9.56)	16.50 (10.15)	13.79 (9.63)	17.79 (10.74)
MF = 1/[1+(Age/TM_50_)^−*γ*^]							
TM_50_ (SE%)	–	–	–	0.90 (26.43)	–	–	–
γ (SE%)	–	–	–	0.08 (31.64)	–	–	–
k_1_	0.75	0.79 (11.64)	1.03 (11.39)	–	–	–	–
$k_{1}=k_{0}-k_{\max}\times WT^{\gamma }/\left(k_{50}^{\gamma }+WT^{\gamma }\right)\text{ or }k_{1}=k_{0}-k_{\max}\times {\text{Age}}^{\gamma }/\left(k_{50}^{\gamma }+{\text{Age}}^{\gamma }\right)\text{ or }k_{1}\;=\;k_{0}-k_{\max}\times {\text{BSA}}^{\gamma }/\left(k_{50}^{\gamma }+{\text{BSA}}^{\gamma }\right)$							
k_0_ (SE%)	–	–		–	2.18 (25.46)	1.42 (14.62)	1.60 (27.51)
k_max_ (SE%)	–	–		–	1.52 (21.27)	0.95 (29.45)	0.38 (30.23)
k_50_ (SE%)	–	–		–	4.15 (20.61)	0.35 (39.51)	0.56 (36.61)
γ (SE%)	–	–		–	16.97 (44.52)	0.44 (33.09)	1.08 (37.58)

WT, body weight; BSA, body surface area; OFV, objective function value; AIC, Akaike information criterion; BIC, Bayesian information criterion; θ_CL_, typical value of clearance; θ_Vd_, typical value of volume of distribution; SE, standard error; MF, factor for maturation; TM_50_, maturation half-time; γ, Hill coefficient defining the steepness of the sigmoidal curve; k^1^, allometric exponent; k_0_, the exponent at a theoretical weight of 0 kg , BSA of 0 m^2^, or age at 0 years; k_max_, a maximum decrease of the exponent; k_50_, the weight, BSA or age when a 50% drop in the maximum decrease of the exponent is achieved.

**TABLE 4:** Covariate screening and final model development process

Steps	Covariates screening	OFV	ΔOFV	*p* value	Comments
1	None forward inclusion	359.92			Base model
2	CL-WT	301.70	−58.22	<0.01	
3	CL-WT/V_d_-WT	293.65	−8.05	<0.01	
4	CL-WT-KF/V_d_-WT	285.73	−7.92	<0.01	
5	CL-WT-KF-*ALT*/V_d_-WT backward elimination	280.89	−4.84	<0.05	Full model
6	CL-WT-KF/V_d_-WT	285.73	4.84	>0.01	Final model

OFV, objective function value; ΔOFV, change of objective function value; CL, apparent oral clearance; V_d_, apparent volume of distribution; WT, body weight; KF, kidney function; ALT, alanine aminotransferase concentration.

The final model for parameter estimation is presented as follows:

$$V_{d}\left(L\right)=\theta_{V_{d}}\times {\left(\frac{WT}{12.0}\right)}^{\theta_{1}}$$ (5)

$$CL\left(L\cdot h^{-1}\right)=\theta_{CL}\times KF^{\theta_{2}}\times {\left(\frac{WT}{12.0}\right)}^{\theta_{3}}$$ (6)

where *V*_d_ is the apparent volume of distribution, CL is the apparent oral clearance, WT is body weight, and KF is kidney function.

**FIGURE 3:**
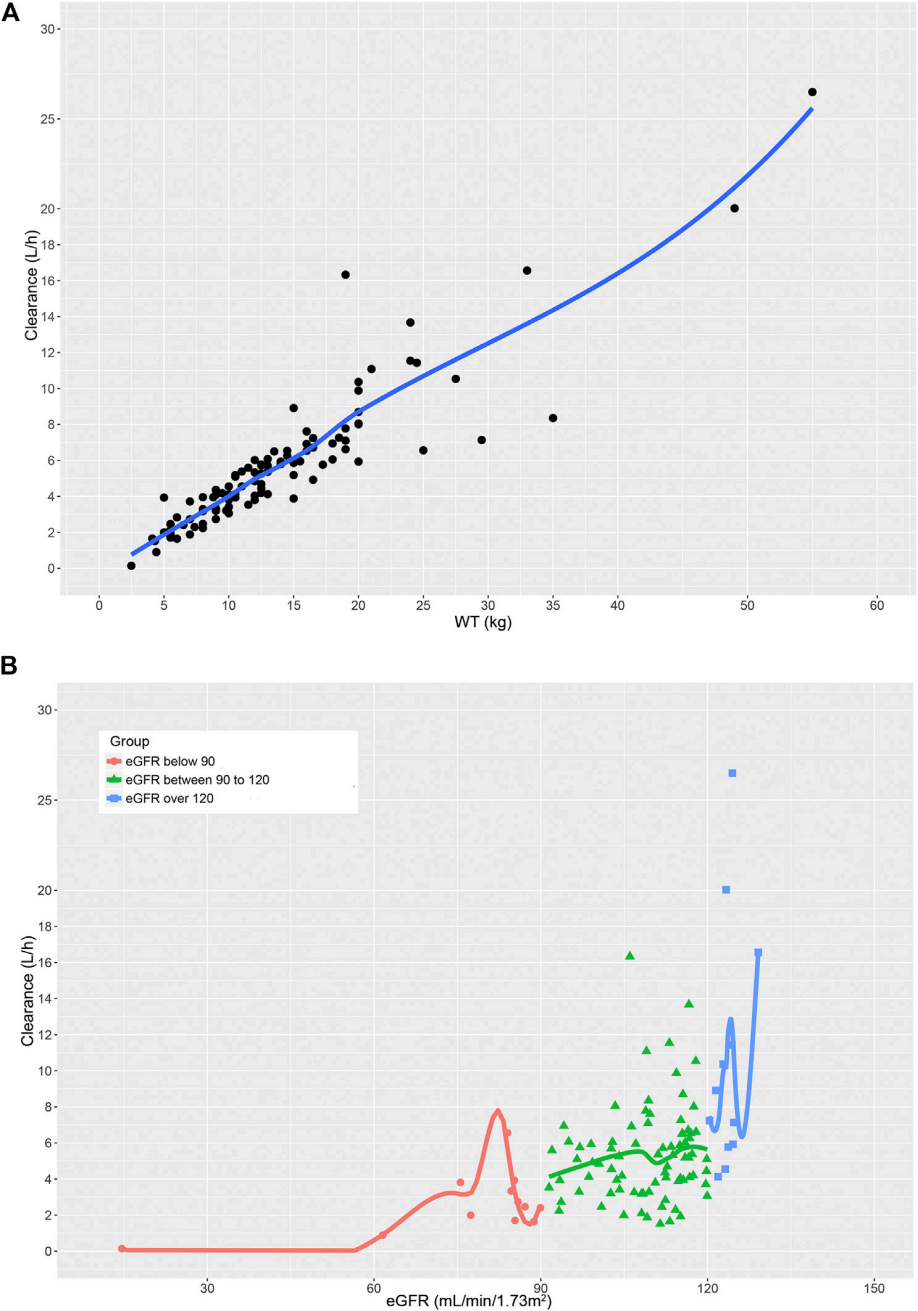
The relationship between **(A)** GCV clearance and WT. **(B)** GCV clearance and eGFR in children with different renal function.

The pharmacokinetic parameter estimates of the final model are presented in Table 5. The typical value of *V*_d_ and CL were 11.35 and 5.23 L/h, respectively. The individual Bayesian estimates of CL was 0.40 ± 0.10 L/h/kg. The relationships between significant variables and CL were depicted by the locally weighted scatterplot smoothing (LOWESS) curves (Figure 3). Noteworthy, the result of the one-way ANOVA test showed significant differences in CLs among the three groups (*p* = 0.010, F = 4.804).

**TABLE 5:** Pharmacokinetic parameter estimates from the final model and bootstrap results.

Parameter	Final model		Bootstrap analysis			Bias^a^ (%)
	Estimate	SE (%)	Median estimate	2.5th Percentile	97.5th Percentile	
θ_*V*d_ (L)	11.35	9.77	11.26	8.43	14.00	−0.79
θ_CL_ (L·h^−1^)	5.23	6.60	5.17	4.12	5.95	−1.15
θ_1_	0.80	19.52	0.79	0.44	1.14	−1.25
θ_2_	0.92	21.77	0.97	0.48	1.46	5.43
θ_3_	1.02	11.63	0.98	0.58	1.32	−3.92
Inter-individual						
*ω*_*V*d_ (%)	65.78	19.20	63.79	38.31	89.27	−3.03
*ω*_CL_ (%)	12.90	38.06	13.11	3.30	22.91	1.63
Residual variability						
*σ* (%)	8.23	23.61	8.37	4.12	13.37	1.70

SE, standard error; θ_Vd_, typical value of apparent volume of distribution; θ_CL_, typical value of apparent clearance; θ_1_, exponent for WT as covariate for V_d_; θ_2_, exponent for KF as covariate for CL; θ_3_, exponent for WT as covariate for CL; ω_Vd_, square root of interindividual variance for V_d_; ω_CL_, square root of interindividual variance for CL; σ, residual variability.

^a^ Bias = (median estimate from bootstrap analysis—estimate from the final model)/estimate from the final model.

### Model Evaluation

**FIGURE 4:**
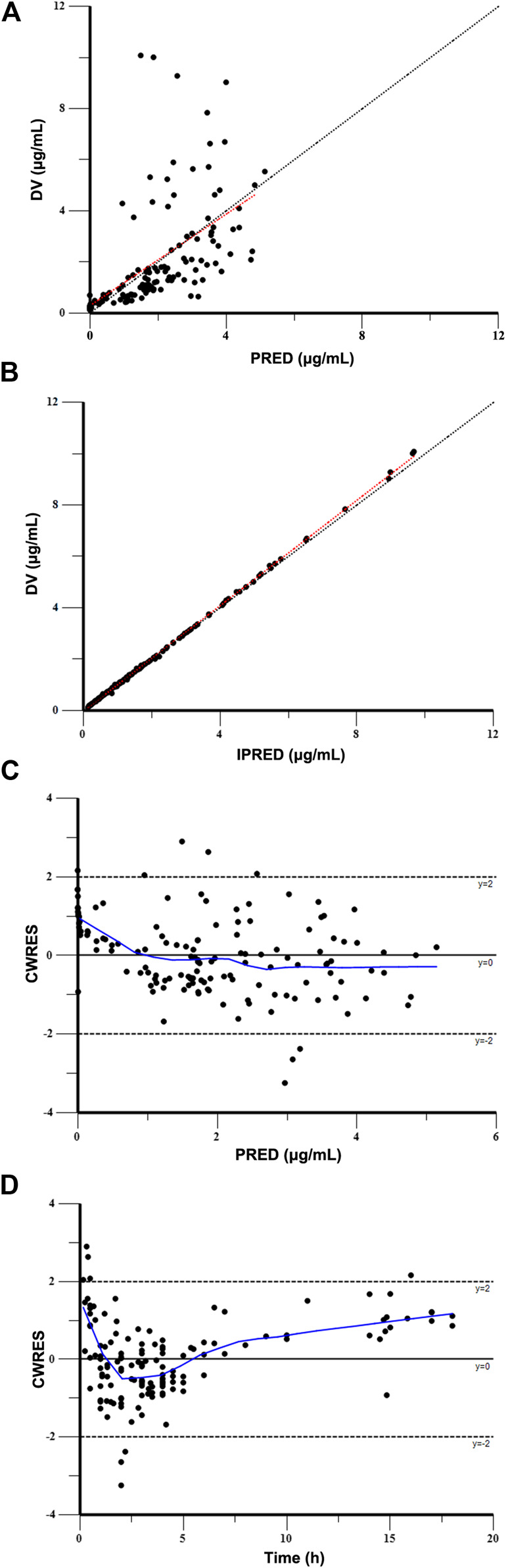
Goodness-of-fit plot for the final model. **(A)** Observed concentrations (DV) vs. population predictions (PRED), **(B)** DV vs. individual predictions (IPRED), **(C)** conditional weighted residuals (CWRES) vs. PRED, and **(D)** CWRES vs. time.

**FIGURE 5:**
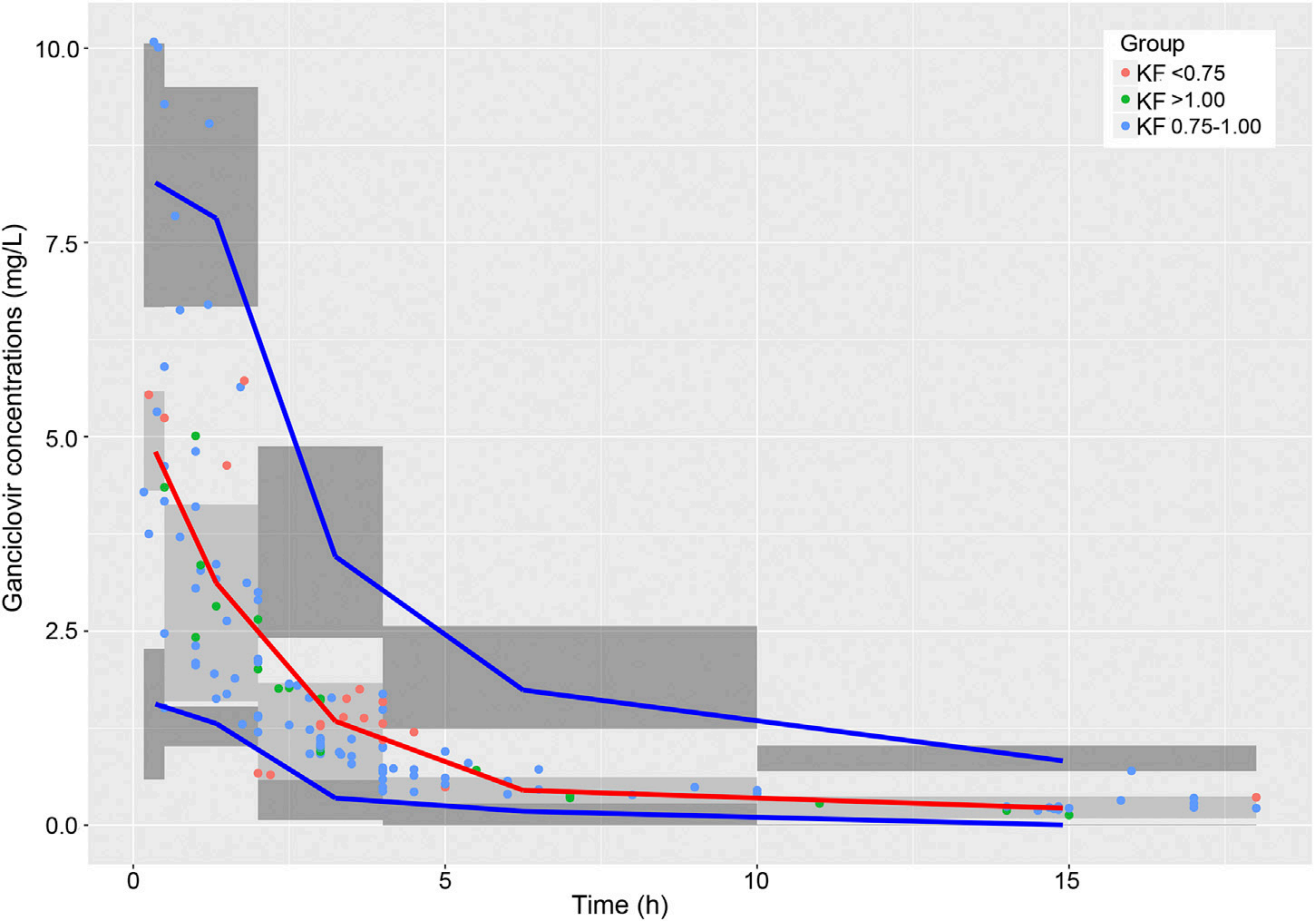
Visual predictive check of the final model. The observed concentrations in patients with different renal functions are shown as red, blue, and green circles. The red line is the 50th percentile of the simulated data, and lower and upper blue lines represent the 2.5th and 97.5th percentiles of the simulated data, respectively. The three shaded areas represent the 95% intervals of the 2.5th, 50th and 97.5th percentiles of the simulated concentrations, respectively.

**FIGURE 6:**
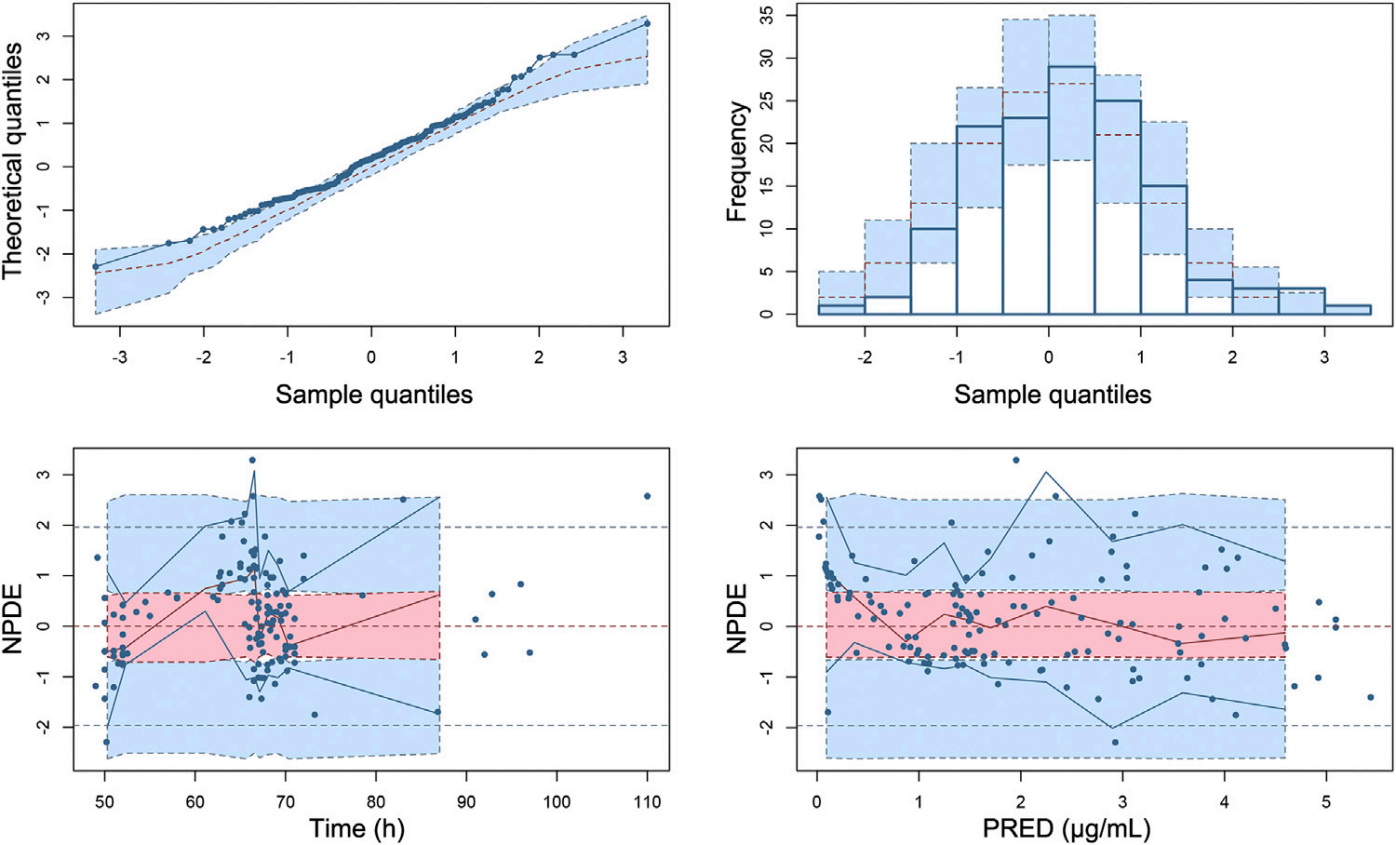
Normalized prediction distribution errors (NPDE) of the final population pharmacokinetic model. **(A)** Quantile–quantile plot of NPDE vs. the expected standard normal distribution, **(B)** histogram of NPDE with the density of the standard normal distribution overlaid, **(C)** Scatterplot of NPDE vs. time, and **(D)** scatterplot of NPDE vs. PRED. Blue dots represent observed concentrations. Red lines show the medians of observed data and blue lines show the 5th and 95th percentiles of observed concentrations. Red or blue shaded areas represent the 95% prediction interval for the respective metric.

The Goodness-of-fit plots for the final model showed that the predictions were found to be in acceptable agreement with the observations. The majority of the conditional weighted residuals were between −3 to +3 (Figure 4). Furthermore, the values of model estimates were similar to that of bootstrap median estimates with a slight bias of lessing than ±8%. All parameter estimates from the final model were included in 95% CI computed from bootstrap analysis (Table 5). The VPC plots showed that most observations were positioned within the 95% CI of the simulations (Figure 5), indicating the good prediction performance of the final model. No obvious trend was observed in the scatterplots for NPDE analysis (Figure 6). Besides, the *p* values were 0.122, 0.623, 0.289, and 0.367 obtained from the Wilcoxon signed rank test, the Fisher test for variance, the Shapiro-Wilks test, and the global test, respectively. The results confirmed that the NPDE exhibited homogeneity of variance and also conformed to a normal distribution.

### Dosing Simulations

**FIGURE 7:**
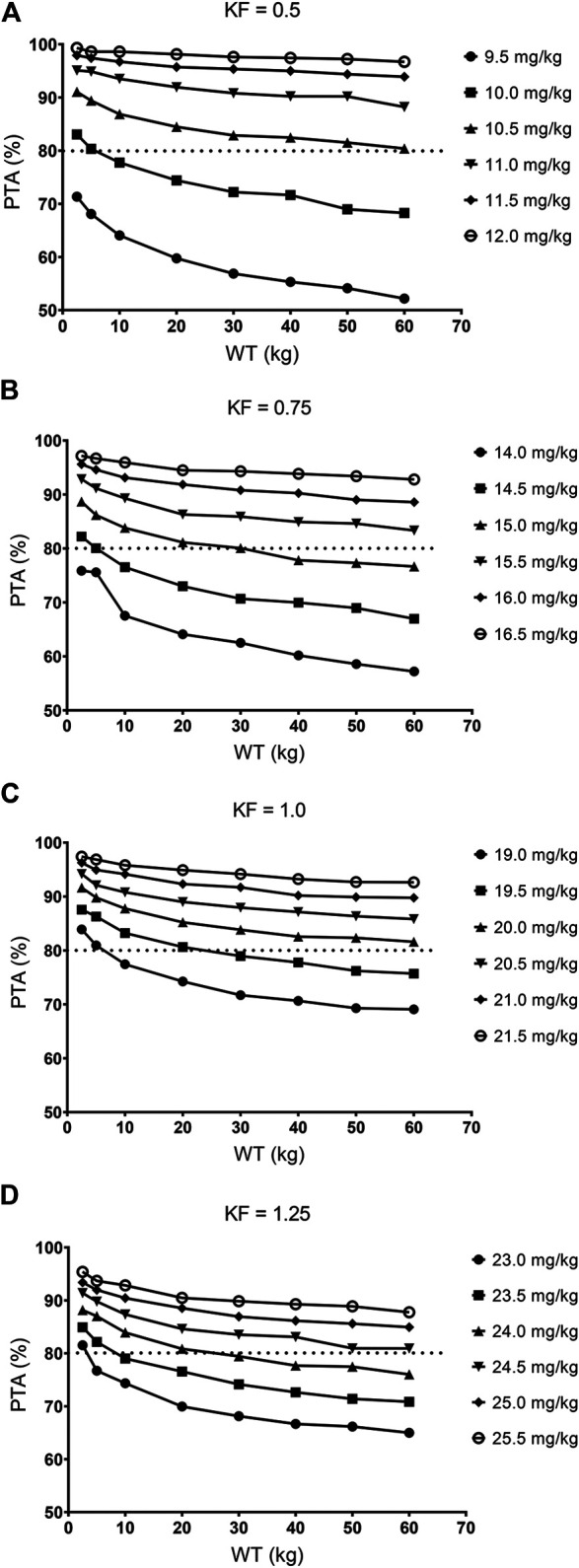
The PTAs of various dosing regimens for patients with different WT levels.

Table 6 and Figure 7 showed the PTA values of different dosing regimens for patients with various renal function status and WT levels. The results of Monte Carlo simulations demonstrated that the current clinical dosage (10 mg/kg/d) was associated with insufficient drug exposure and resulted in pretty low PTA values for patients who weighed more than 5 kg in all renal function groups. When GCV was dosed on a linear WT adjusted basis (mg/kg), dosing regimens of 15.0, 20.0, and 21.0 mg/kg/d provided acceptable PTAs in patients with mild renal insufficiency (83.37%), normal renal function (85.68%), and elevated renal function (82.15%), respectively. On the other hand, when KF was fixed at 0.5, 0.75, 1.0, and 1.25, adequate PTA could be achieved in dosage regimens of 10–10.5, 14.5–15.5, 19.0–20.0, and 23.0–24.5 mg/kg/d, respectively (Table 7).

**TABLE 6:** The PTAs of different dosing regimens for patients with varying renal function

	Dose (mg/kg/d)	AUC_0–24_		
Classes of renal function		Mean (μg·h/ml)	Range	PTA (%)
60–90 mL/min/1.73 m^2^ (KF 0.5–0.75)	10.0	32.55	15.11–69.79	11.36
	12.5	40.79	19.91–74.21	50.25
	15.0	48.81	22.76–99.30	83.37
	17.5	56.96	26.91–111.60	96.19
90–120 mL/min/1.73 m^2^ (KF 0.75–1.0)	10.0	25.54	10.56–49.18	0.92
	15.0	38.24	16.17–80.18	37.48
	20.0	51.03	24.25–104.71	85.68
	25.0	63.54	26.73–136.86	98.39
> 120 mL/min/1.73 m^2^ (KF>1.0)	10.0	22.98	11.62–43.30	0.40
	15.0	34.62	15.96–69.57	19.45
	20.0	46.10	22.27–87.61	74.63
	21.0	48.41	23.36–97.15	82.15
	25.0	57.76	27.98–108.64	96.80

AUC_0-24_, the area under drug plasma concentration-time curve over 24 h; PTA, the probability of target attainment; KF, kidney function.

**TABLE 7:** Optimal dosage regimens based on simulation.

eGFR or KF	WT (kg)	Dosage regimen (mg/kg/d)
eGFR = 60 mL/min/1.73m^2^ or KF = 0.5	2.5	10.0
	5	10.0
	10	10.5
	20	10.5
	30	10.5
	40	10.5
	50	10.5
	60	10.5
eGFR = 90 mL/min/1.73m^2^ or KF = 0.75	2.5	14.5
	5	14.5
	10	15.0
	20	15.0
	30	15.0
	40	15.5
	50	15.5
	60	15.5
eGFR = 120 mL/min/1.73m^2^ or KF = 1.0	2.5	19.0
	5	19.0
	10	19.5
	20	19.5
	30	20.0
	40	20.0
	50	20.0
	60	20.0
eGFR = 150 mL/min/1.73m^2^ or KF = 1.25	2.5	23.0
	5	23.5
	10	24.0
	20	24.0
	30	24.5
	40	24.5
	50	24.5
	60	24.5

eGFR, estimated glomerular filtration rate; KF, kidney function; WT, body weight.

### Assessment of Adverse Events

Hematological parameters obtained before and after GCV treatment were summarized in Table 8. The mean values of hemoglobin, platelet, and leukocyte did not change after therapy in comparison with the baseline, while only a significant decrease in neutrophil counts was found during GCV treatment (*p* < 0.001). In the current study, 8 cases of grade 2 anemia (7.69%), 2 cases of grade 2–3 lymphopenia (1.92%), and 20 cases of grade 2–4 neutropenia (19.23%) were observed, while no thrombocytopenia was found. Individual values for AUC_0-24_, trough concentration (*C*_min_), peak concentration (*C*_max_), and the time above GCV concentration of 0.025–1.5 μg/mL (Tc > 0.025–1.5 μg/mL) were derived using Bayesian method. The relationship between systemic exposure and incidence of neutropenia was analyzed using multivariate logistic regression. Both unadjusted odds ratio (OR) and adjusted OR were estimated. Furthermore, all patients were stratified by different trough concentration levels, and the incidence of neutropenia between groups were compared by Pearson Chi-square tests. The results were shown in Supplementary Material Tables S1 and S2. The *p*-values obtained through statistical analyses implied that the GCV exposure (*C*_min_, *C*_max_, AUC_0-24_, Tc > 0.025–1.5 μg/mL) had no significant influence on the occurrence of neutropenia under current dosage regimen (10 mg/kg/d).

**TABLE 8:** Hematological parameters before and after GCV treatment.

	Before GCV treatment	After GCV treatment	*p* value
Hemoglobin (g L^−1^)	109.7 ± 17.0	110.1 ± 14.9	0.815
Platelets (10^9^/L)	264.6 ± 132.4	268.1 ± 121.6	0.699
Lymphocytes (10^9^/L)	5.00 ± 2.90	4.77 ± 2.74	0.193
Neutrophils (10^9^/L)	5.01 ± 4.44	3.13 ± 2.18	<0.001

GCV, ganciclovir.

## Discussion

The majority of the current PopPK studies for GCV was focused on neonates with congenital CMV infection, pediatric and adult solid organ transplant patients. The detailed data on GCV pharmacokinetics in critically ill children was almost blank. Therefore, our study attempted to fill the research gaps concerning the pharmacokinetic profiles and dose individualization of GCV in this population.

In the present study, the disposition kinetics of GCV in critically ill children was adequately described using a one-compartment model with first-order elimination. WT and eGFR were found to have significant effects on GCV clearance. When normalized for WT, the Bayesian estimates of CL (0.40 ± 0.10 L/h/kg) was in line with the one reported in pediatric renal transplant recipients (0.39 ± 0.14 L/h/kg) (Facchin et al., 2019), while slightly higher than the CL reported in neonates with congenital CMV disease (0.287 L/h/kg) (Acosta et al., 2007). This discrepancy may be attributed to physiologic changes in clearance processes that occurred during childhood development.

As shown in the covariate analysis results, both WT and KF were identified as the most influential parameters on CL. It is well recognized that the most obvious difference between children and adults is the body size (Holford et al., 2013), which is generally parameterized by WT or BSA. Our study demonstrated that WT as a primary covariate was superior to BSA when allometric exponent model was used, which was inconsistent with the Jorga et al. (2019). In addition, considering that GCV is a renally excreted antiviral drug with high hydrophilicity, the renal function would significantly alter the clearance capacity of GCV (Tsai et al., 2015). KF and eGFR were determined to reflect the renal function and also had an important influence on the prediction of CL. It was evident from the LOWESS curves that WT showed a significant positive correlation with CL, while only a weak positive relationship between eGFR and CL was observed. Intriguingly, although there existed significant differences in CL among the three renal function groups, this statistical significance disappeared after WT-normalization of CL. This could be elucidated by the fact that the number of cases in mild renal insufficiency group and elevated renal function group was small.

Currently, various dosing algorithms for intravenous ganciclovir have been proposed in published researches. And WT, BSA, eGFR or creatinine clearance values were used to compute individual GCV doses (Jorga et al., 2019). According to the result of the covariate analysis, the WT-based algorithm was considered more appropriate than the BSA-based algorithm for dosage regimen design for critically ill pediatric patients. For renally excreted drugs, the recommended standard dose may be inadequate for patients with elevated renal function, which ultimately resulted in therapy failure or drug resistance in this population. After comprehensive consideration, patients in our study were stratified according to different renal function status and WT levels for simulation-based dosage evaluation and optimization. The simulation results suggested that the commonly used dosing regimen (10 mg/kg/d) would lead to underexposure for nearly all patients in three renal function groups. Therefore elevated doses might be required to achieve therapeutic pharmacodynamic targets. Furthermore, WT and renal function based approach could be used to individualize GCV dosing and to promote clinical efficacy.

It was reported that the main adverse effect related to GCV treatment was hematologic toxicity, including which the incidence of neutropenia was the most common (Kimberlin, 2002). Similarily, neutropenia was also demonstrated to be the most common adverse effects in the present study (18.27%). However, we couldn’t find definite association between GCV exposure and the incidence of neutropenia when a conventional dose of GCV (10 mg/kg/d) was given. However, the link between exposure to GCV and the occurrence of neutropenia is still controversial (Paya et al., 2004; Wiltshire et al., 2005; Billat et al., 2016). Wiltshire et al. (2005) proposed that higher GCV exposure might result in a tendency to increased neutropenia. In contrast, Billat et al. (2016) found that the decrease in the neutrophil count was associated with intracellular GCV triphosphate exposure rather than plasma GCV level during treatment. In this regard, further studies were needed to obtain more compelling evidence.

There are several limitations in this study. Firstly, external validation could not be implemented due to the small population size. Secondly, a low proportion of patients with renal insufficiency making it difficult to establish a clear link between renal function and pharmacokinetic parameters of GCV. Finally, pharmacokinetic-pharmacodynamic relationship cannot be investigated owing to the lack of clinical data.

## Conclusion

In summary, a PopPK model for intravenous GCV in children suffered from critical illness had been successfully established and validated. Results showed that only WT and KF were considered to be major determinants of GCV pharmacokinetic parameters. For critically ill pediatric patients, the recommended clinical dosage (10 mg/kg/d) could lead to a high risk of underexposure. And elevated doses might be required to reach target GCV exposure and improve therapeutic effect in this vulnerable population. Futhermore, the model-based simulations also demonstrated that GCV dosing based on WT and renal function was rational. It's worth noting that the association between high-doses GCV and risk of adverse events is still unclear, therefore high doses of GCV should be used with extra attention.

## Data Availability Statement

The original contributions presented in the study are included in the article/Supplementary Material, further inquiries can be directed to the corresponding author.

## Ethics Statement

The studies involving human participants were reviewed and approved by The Ethics Committee of Wuhan Children’s hospital. Written informed consent from the participants’ legal guardian/next of kin was not required to participate in this study in accordance with the national legislation and the institutional requirements.

## Author Contributions

SL drafted the manuscript. YW, SW, and HX concepted and supervised the study. SL and YW contributed to the analysis and interpretation of the data. SL and CS contributed to study conduction, data acquisition, and critical revision of the manuscript.

## Funding

This study is supported by Wuhan Municipal Health Commission scientific research project (Grant Agreement Number WX18C21) and the Youth Program of the National Natural Science Foundation of China (Grant Agreement Number 81600123). The authors declare that they have no conflicts of interest in relation to this work.

## Conflict of Interest

The authors declare that the research was conducted in the absence of any commercial or financial relationships that could be construed as a potential conflict of interest.
