# Supplementary material for: Population Pharmacokinetics and Dose Optimization of Ganciclovir in Critically Ill Children
Source: Front Pharmacol. 2021 Jan 18;11:614164. doi: 10.3389/fphar.2020.614164 (PMC7847843; doi:10.3389/fphar.2020.614164)
Supplement: Supplementary file 1 [file datasheet1.pdf]

Supplementary material

Table S1 Multivariate logistic regression analyzing the influence of GCV exposure on the incidence of neutropenia

Parameters	Crude OR (95% CI)	*p*-value	Adjusted OR[a] (95% CI)	*p*-value
C_{min}	0.833(0.262-2.646)	0.756	0.798(0.254-2.504)	0.699
C_{max}	0.912(0.733-1.135)	0.409	0.918(0.735-1.146)	0.449
AUC_{0-24}	1.033(0.957-1.116)	0.401	1.038(0.958-1.125)	0.359
Tc >0.025 μg/mL	1.170(0.926-1.480)	0.189	1.170(0.923-1.485)	0.195
Tc >0.1 μg/mL	1.099(0.919-1.314)	0.300	1.097(0.915-1.314)	0.317
Tc >0.2 μg/mL	1.071(0.905-1.266)	0.425	1.066(0.900-1.264)	0.459
Tc >0.5 μg/mL	1.102(0.905-1.343)	0.333	1.102(0.901-1.347)	0.344
Tc >1.0 μg/mL	1.210(0.881-1.663)	0.239	1.209(0.869-1.682)	0.260
Tc >1.5 μg/mL	1.037(0.854-1.261)	0.712	1.027(0.840-1.256)	0.797

[a] *Adjusted by gender, age, and body weight.*

OR, odds ratio; C_{min}, trough concentration;C_{max}, peak concentration, AUC_{0-24}, the area under drug plasma concentration-time curve over 24 hours; Tc >0.025-1.5 μg/mL, the time above GCV concentration of 0.025 to 1.5 μg/mL.

Table S2 Chi-square tests analyzing the effects of different trough concentration levels on the incidence of neutropenia

C_{min} (μg/mL)	No neutropenia (n=84)	Neutropenia (n=20)	χ^2	*p*-value
>0.01	54	15	0.831	0.362
>0.02	47	14	1.314	0.252
>0.025	43	14	2.307	0.129
>0.03	43	13	1.240	0.266
>0.05	38	11	0.618	0.432
>0.1	30	7	0.004	0.952
>0.15	22	5	0.012	0.913
>0.3	13	4	0.242	0.623
>0.5	5	2	0.422	0.516

C_{min}, trough concentration.
